# CPLM: a database of protein lysine modifications

**DOI:** 10.1093/nar/gkt1093

**Published:** 2013-11-08

**Authors:** Zexian Liu, Yongbo Wang, Tianshun Gao, Zhicheng Pan, Han Cheng, Qing Yang, Zhongyi Cheng, Anyuan Guo, Jian Ren, Yu Xue

**Affiliations:** ^1^Department of Biomedical Engineering, College of Life Science and Technology, Huazhong University of Science and Technology, Wuhan, Hubei 430074, China, ^2^Advanced Institute of Translational Medicine, Tongji University, Shanghai 200092, China and ^3^State Key Laboratory of Biocontrol, School of Life Sciences, Sun Yat-sen University, Guangzhou, Guangdong 510275, China

## Abstract

We reported an integrated database of Compendium of Protein Lysine Modifications (CPLM; http://cplm.biocuckoo.org) for protein lysine modifications (PLMs), which occur at active ε-amino groups of specific lysine residues in proteins and are critical for orchestrating various biological processes. The CPLM database was updated from our previously developed database of Compendium of Protein Lysine Acetylation (CPLA), which contained 7151 lysine acetylation sites in 3311 proteins. Here, we manually collected experimentally identified substrates and sites for 12 types of PLMs, including acetylation, ubiquitination, sumoylation, methylation, butyrylation, crotonylation, glycation, malonylation, phosphoglycerylation, propionylation, succinylation and pupylation. In total, the CPLM database contained 203 972 modification events on 189 919 modified lysines in 45 748 proteins for 122 species. With the dataset, we totally identified 76 types of co-occurrences of various PLMs on the same lysine residues, and the most abundant PLM crosstalk is between acetylation and ubiquitination. Up to 53.5% of acetylation and 33.1% of ubiquitination events co-occur at 10 746 lysine sites. Thus, the various PLM crosstalks suggested that a considerable proportion of lysines were competitively and dynamically regulated in a complicated manner. Taken together, the CPLM database can serve as a useful resource for further research of PLMs.

## INTRODUCTION

In 1964, Allfrey *et al.* (1) first observed gene expression regulation mediated by covalently introducing acetyl and methyl groups on lysine residues in histones. Numerous following studies in epigenetics proposed the combinational post-translational modifications (PTMs) of histones as ‘histone codes’, of which PTMs occurring on lysine residues occupy an important proportion (2). Later studies discovered lysine as a hot spot for PTMs, while a number of protein lysine modifications (PLMs) can occur in both histone and non-histone proteins (3–11). For example, beyond constituting the ‘histone code’, lysine acetylation plays a critical role in various biological processes such as metabolism (12,13) and autophagy (14,15), while methylation in non-histone proteins can regulate protein stability and activity (16). In 2004, the Nobel Prize in Chemistry was awarded to Aaron Ciechanover, Avram Hershko and Irwin Rose for their discovery of ubiquitin conjugation on lysine as a mechanism that targets proteins for degradation (17). Also, ubiquitin-like proteins such as small ubiquitin-related modifier and prokaryotic ubiquitin-like protein were found to modify protein lysine residues through a conserved conjugation cascade (18,19). In addition, protein lysines can be modified to 3-phosphoglyceryl-lysine by the primary glycolytic intermediate 1,3-bisphosphoglycerate (1,3-BPG) (10), whereas lysine glycation is involved in glycolytic processes (11).

Recently, rapid progresses in proteomic technologies greatly advanced the identification of well-characterized PLMs (20–23) and the discovery of new PLMs (4,6–8,10). For example, with a monoclonal antibody for diglycine (diGly)-containing isopeptides, Kim *et al.* (21) identified and quantified nearly 20 000 ubiquitination sites. Also, Udeshi *et al.* (22) refined a preparation procedure and used anti-diGly antibodies to quantify ∼20 000 ubiquitination sites. In 2012, Lundby *et al.* (23) quantified ∼15 000 acetylation sites from 16 rat tissues and systematically analyzed the tissue-specific lysine acetylation profiles. In particular, with the state-of-the-art proteomic techniques, Dr. Yingming Zhao’s group has identified a number of new PLMs such as butyrylation (4), propionylation (4), malonylation (6), crotonylation (7) and succinylation (8). Because the numbers of PLMs and modified lysine residues have been greatly expanded, an integrated resource for the community is urgently needed. Although several public databases such as UniProt (24), HPRD (25), SysPTM (26) and dbPTM (27) contained information for PLMs, only a limited proportion of the identified substrates and sites were covered, and the newly discovered PLMs were not considered.

Previously, we developed the Compendium of Protein Lysine Acetylation (CPLA) database to maintain the identified lysine acetylation information (28). In this work, we greatly improved the CPLA database by extending the types of PLMs and developed the database of Compendium of Protein Lysine Modifications (CPLM). From scientific literature, the experimentally identified substrates and sites for 12 types of PLMs were manually collected. Besides acetylation, well-studied PLMs such as ubiquitination, sumoylation, methylation and glycation and newly discovered PLMs including butyrylation, crotonylation, malonylation, phosphoglycerylation, propionylation, succinylation and pupylation were integrated into the database. Currently, CPLM database contained 203 972 modification events on 189 919 modified lysine residues in 45 748 proteins from 122 species, and the detailed annotations were also provided. The database can be searched or browsed in a convenient manner. Based on the comprehensive dataset, we systematically analyzed the concurrences of different PLMs at the same lysine residues. Although the number of identified substrates and sites for different types of PLMs varies from ten thousands to tens, each PLM can crosstalk with at least one other PLM and the co-occurrences of different PLMs at the same site were particularly abundant. From 76 types of identified PLM co-occurrences, we observed that the crosstalks among acetylation, ubiquitination and succinylation are mostly abundant. The intensive crosstalks among PLMs suggested that at least a considerable number of lysines were competitively and dynamically regulated by different PLMs. Taken together, the CPLM database provided an integrative platform for the community to access the current processes on PLMs and generated a useful resource for further experimental or computational considerations. The CPLM database was implemented in PHP + MySQL + JavaScript.

## CONSTRUCTION AND CONTENT

As previously described (28), we searched PubMed with keywords including ‘acetylation’, ‘ubiquitination’, ‘sumoylation’, ‘methylation’, ‘glycation’, ‘butyrylation’, ‘crotonylation’, ‘malonylation’, ‘phosphoglycerylation’, ‘propionylation’, ‘succinylation’ and ‘pupylation’ and manually curated literature to collect the experimentally identified PLM substrates and sites. To avoid missing data, additional keywords such as ‘acetylated’, ‘acetyl’, ‘ubiquitinated’ and other related nomenclatures were employed for searching more data in PubMed. All modified lysine residues were mapped to the benchmark sequences retrieved from the UniProt database (Release 2013_08) (24). To provide more information for the PTMs substrates, the annotations from UniProt (24) were integrated into the database. The primary references for PLM substrates and sites were also provided to ensure the quality of the database.

**Figure 1.**
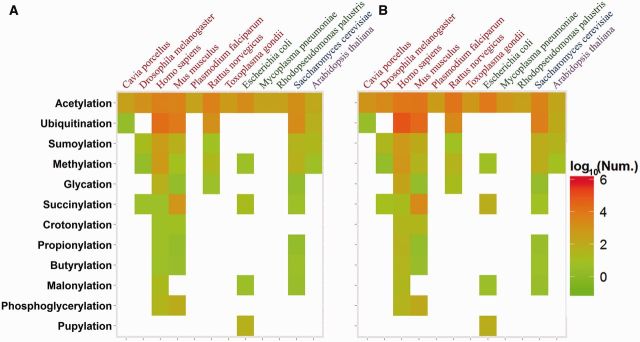
The heatmaps for the protein number distribution of different PLM types and species. The species names in red, green, blue and purple are from animals, bacteria, fungi and plants, respectively. (**A**) The heatmap for the number of substrates; (**B**) the heatmap for the number of modified lysine residues.

In total, 203 972 modification events were found to occur on 189 919 lysine residues in 45 748 substrates for 12 types of PLMs (Supplementary Table S1). Obviously, acetylation and ubiquitination have the most substrates; the former contains 58 563 sites in 20 088 proteins and the latter contains 139 950 sites in 32 429 proteins (Supplementary Table S1). The third PLM with most substrates is succinylation (8), which was discovered as a novel PLM in 2011 and identified with 2523 sites in 897 substrates (Supplementary Table S1). The rapid progress in the identification of succinylation is attributed to the advancement of proteomic techniques (29). However, for other new PLMs such as butyrylation, crotonylation, malonylation, phosphoglycerylation and propionylation, there were only a small number of identified substrates that mainly focused on histones (Supplementary Table S1). Although various PLMs were experimentally detected in 122 species, the number of identified substrates is usually limited for most organisms. With the ggplot2 program (30) in the R package (31), the distribution of PLM substrates and sites from 12 major species with >200 substrates were visualized (Figure 1A and B). Clearly, animals, especially mammals, were identified with most substrates (Figure 1A) and sites (Figure 1B). It is worthy to note that several types of PLMs are only exclusively identified in distinct species. For example, ubiquitination and sumoylation are only available in eukaryotes, while pupylation was only discovered in actinomyces.

## USAGE

**Figure 2.**
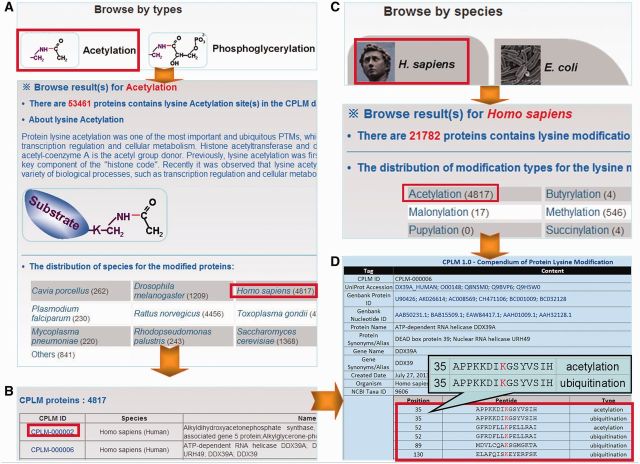
The browse options of CPLM. Two browse approaches including by PLM types and by species were provided to browse the database. (**A**) By PLM types; (**B**) the protein list for specified PLM and selected organism; (**C**) by species; (**D**) the detailed information of human dead box protein 39.

The CPLM database was developed in a user-friendly manner, while browse and search options were provided for accessing the information. Because the proteins and sites could be classified according to the PLM types and species, two browse options including ‘Browse by types’ and ‘Browse by species’ were developed in the database (Figure 2). For convenience, only 12 major species were listed for browsing, while all the other organisms were denoted as ‘Others’. Here, we use lysine acetylation substrates from *Homo sapiens* as an example to present the usage of the browse options in CPLM. In the option of ‘Browse by types’, 12 simplified molecular structures of ligands conjugated to lysine residues during modification were employed to represent the 12 types of PLMs (Figure 2A). By clicking on the ‘Acetylation’ button, a brief introduction of protein lysine acetylation and the protein number distribution of acetylated proteins in 12 major organisms and other species were showed (Figure 2A). Then the acetylation substrates in *H. sapiens* could be listed through clicking on the ‘*Homo sapiens*’ link (Figure 2B). In the option of ‘Browse by species’, the 12 major organisms were organized as animals, bacteria, fungi and plants. Users could click on the ‘*H. sapiens*’ button to view the protein number distribution of different PLM substrates in *H. sapiens* (Figure 2C), and then click on the link of ‘Acetylation’ to view the list of acetylated substrates in *H. sapiens* (Figure 2B). The detailed information for any specified protein could be accessed through the links in the list (Figure 2D).

**Figure 3.**
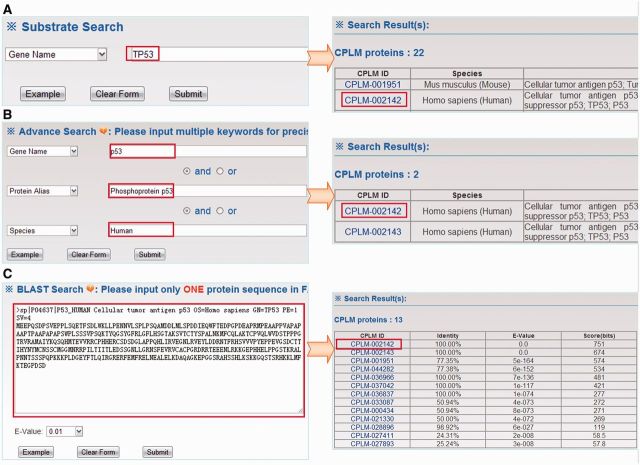
The search options. (**A**) The database could be queried with simple keywords input; (**B**) the ‘Advance Search’ allows users to submit combination of up to three terms for searching; (**C**) the database could be queried with a protein sequence to find identical or homologous proteins.

For convenient usage, three search options were implemented for querying the database with one or multiple keywords. For example, if users search the keyword ‘TP53’ in the ‘Gene Name’ area, the results will be shown in a tabular format with CPLM ID, organism and protein/gene names/aliases (Figure 3A). Furthermore, two options including ‘Advance Search’ and ‘BLAST Search’ were developed to query the proteins with higher accuracy. In the ‘Advance Search’ option, users can submit up to three search terms, which could be specified in different areas and combined with three operators of ‘and’, ‘or’ and ‘exclude’ to perform a complex query (Figure 3B). The ‘BLAST search’ option was designed to find similar proteins with a protein sequence in the FASTA format. Through the application of NCBI BLAST packages (32), users could submit a protein sequence in the FASTA format to search identical or homologous proteins (Figure 3C).

## DISCUSSION

As an important molecular mechanism, PTMs greatly expand the proteome complexity and play a critical role in the regulation of various biological processes (20,33). With the active ε-amino groups, lysine residues were modified by various PLMs, which constitute an important proportion among the large number of PTM types (3). Through modifying the substrates, PLMs regulate various biological processes, while aberrances of lysine modifications were associated with diseases and cancers (18,34–36). Recent development of proteomic techniques greatly advances the identification of PLM substrates and the discovery of new types of PLMs (3,20). However, in contrast to other PTMs such as phosphorylation (37,38), the computational resources for PLMs are still limited.

**Figure 4.**
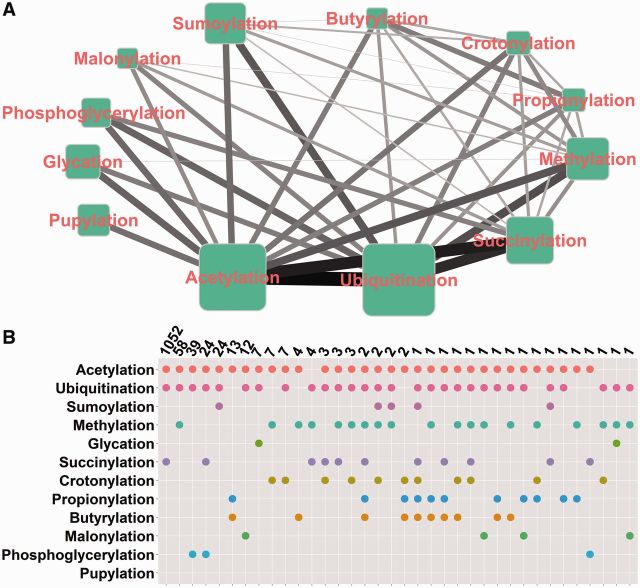
The summary for the number of concurrent sites among different types of PTMs. (**A**) The concurrences between two types of PLMs while the detailed numbers are provided in Supplementary Table S2; (**B**) the concurrences among multiple (>2) types of PLMs while the detailed number are shown in the top.

In this work, we updated the acetylation-associated database of CPLA into CPLM for more types of PLMs. Because 203 972 modification events for 12 types of PLMs were identified on 189 919 lysine residues, it was expected that there were a large number of co-occurrences among different PTMs. Indeed, Weinert *et al.* (39) discovered that the crosstalks between acetylation and succinylation are extensive in both prokaryotes and eukaryotes. Also, previous studies identified that the competition between acetylation and ubiquitination can serve as a mechanism to control protein stability (40) and activity (41). From the data set, we totally identified 76 types of PLM co-occurrences at same lysine residues, including 40 types of pairwise crosstalks (Figure 4A) and 36 types of multiple (three or more) crosstalks (Figure 4B). We observed that the pairwise crosstalks among acetylation, ubiquitination and succinylation are mostly abundant (Figure 4A and Supplementary Table S2). In total, we detected 10 746, 2420 and 1094 pairwise crosstalks for ubiquitination–acetylation, acetylation–succinylation and ubiquitination–succinylation (Supplementary Table S2). Although several PLMs were identified with only a few substrates and sites, each PLM can crosstalk pairwise with at least one other PLM by co-occurring at the same lysines (Figure 4A and Supplementary Table S2). Moreover, the co-occurrences with more than two PLMs at same lysines are also abundant, and the most abundant multiple crosstalk is among acetylation, ubiquitination and succinylation (Figure 4B). Because succinylation is a newly discovered PLM, the functional consequence of crosstalks between succinylation and other PLMs is still not clear. However, it could be anticipated that either pairwise concurrences or multiple crosstalks among acetylation, succinylation and ubiquitination might play a potential role in regulating proteins. In addition, we did not observe co-occurrence with multiple PLMs on pupylated lysine residues (Figure 4B). As a PLM exclusively occurred in actinomyces, pupylation only co-occurs with acetylation in 50 lysine residues (Figure 4A and Supplementary Table S2). The intensive PLM crosstalks suggested that a substantial proportion of lysine residues can be competitively or dynamically regulated by different types of PLMs.

Taken together, here we updated the CPLA database, which only maintained the information of protein lysine acetylation, to CPLM database for an integrated resource of various PLMs. We believed that the updated database can provide a more useful resource for further computational or experimental studies. The CPLM database will be routinely updated to keep pace with the research progresses of PLMs.

## SUPPLEMENTARY DATA

Supplementary Data are available at NAR Online.
